# Season of Data Collection of Child Dietary Diversity Indicators May Affect Conclusions About Longer-Term Trends in Peru, Senegal, and Nepal

**DOI:** 10.1093/cdn/nzab095

**Published:** 2021-07-12

**Authors:** Andrew L Thorne-Lyman, Leah E M Bevis, Helen Kuo, Swetha Manohar, Binod Shrestha, Angela KC, Rolf D Klemm, Rebecca A Heidkamp

**Affiliations:** Center for Human Nutrition, Department of International Health, Johns Hopkins Bloomberg School of Public Health, Baltimore, MD, USA; Center for a Livable Future, Department of Environmental Health and Engineering, Johns Hopkins Bloomberg School of Public Health, Baltimore, MD, USA; Department of Agricultural, Environmental, and Development Economics, The Ohio State University, Columbus, OH, USA; Institute for International Programs, Department of International Health, Johns Hopkins Bloomberg School of Public Health, Baltimore, MD, USA; Center for Human Nutrition, Department of International Health, Johns Hopkins Bloomberg School of Public Health, Baltimore, MD, USA; Policy and Science for Health, Agriculture, and Nutrition Study Team, Johns Hopkins University, Kathmandu, Nepal; Center for Human Nutrition, Department of International Health, Johns Hopkins Bloomberg School of Public Health, Baltimore, MD, USA; Center for Human Nutrition, Department of International Health, Johns Hopkins Bloomberg School of Public Health, Baltimore, MD, USA; Hellen Keller International, New York, NY, USA; Center for Human Nutrition, Department of International Health, Johns Hopkins Bloomberg School of Public Health, Baltimore, MD, USA

**Keywords:** dietary diversity, seasonality, season, Nepal, Peru, Senegal, child diets, dietary quality, indicator

## Abstract

**Background:**

The WHO-UNICEF minimum dietary diversity (MDD) indicator for children aged 6–23 mo is a global monitoring indicator used to track multi-year population-level changes in dietary quality, but the influence of seasonality on MDD estimates remains unclear.

**Objectives:**

To examine how seasonality of data collection may influence population-level MDD estimates and inferences about MDD changes over multiple survey years.

**Methods:**

We selected countries with 3 or more consecutive years of MDD data collection, including continuous national Demographic Health Surveys in Senegal (2012–2017; *n* = 12,183) and Peru (2005–2016; *n* = 35,272) and the Policy and Science for Health, Agriculture, and Nutrition sentinel site seasonal surveys (covering 3 seasons/y) in Nepal (2013–2016; *n*  = 1309). The MDD prevalence (≥5 of 8 food groups) and an 8-item continuous Food Group Score (FGS) and 95% CIs were estimated by month and compared for lean and non-lean seasons using ordinary least squares regression with dummy variables for year.

**Results:**

The national prevalence of MDD was higher in Peru (75.4%) than in Nepal (39.1%) or in Senegal (15.7%). Children in Peru were 1.8% (coefficient, –0.0179; 95% CI, –0.033 to –0.002) less likely to achieve MDD during the lean season. Similar seasonal magnitudes were observed in Senegal (coefficient, –0.0347; 95% CI, –0.058 to –0.011) and Nepal (coefficient, –0.0133; 95% CI, –0.107 to 0.081). The FGS was about 0.1 item lower during the lean season in all 3 countries. In comparison, MDD increased by an average rate of only 4.2 and 4.4 percentage points per 5 y in Peru and Senegal, respectively. Intakes of specific food groups were stable across months in all countries, with the provitamin A–rich food group exhibiting the most seasonality.

**Conclusions:**

The magnitude of seasonal variation in MDD prevalence was smaller than expected but large relative to longer-term changes. If large-scale surveys are not conducted in the same season, biased conclusions about trends are possible.

## Teaser

Seasonality of the minimum dietary diversity indicator for children was explored in Peru, Senegal, and Nepal. If surveys are not conducted in the same season, biased conclusions about trends may be reached.

## Introduction

Policies and interventions focused on improving household diet quality have become a major part of multisectoral nutrition programs in many countries (1). Accordingly, tracking indicators of diet quality for key populations is a growing priority among governments and development partners. The increase in usage of these indicators across sectors is striking: a 2010 review of agriculture projects identified no projects tracking these indicators, but by 2016, the use of diet or food consumption indicators had grown to 93% of 73 surveyed agriculture-to-nutrition projects (2, 3). Simple dietary diversity scores, which tabulate the total number of food groups consumed in a defined recall period (e.g., 24 hours), are of particular interest because they are relatively easy to collect and analyze compared to other diet quality measures and, in the near term, may also be more responsive to programs than anthropometric indicators. Two of the most commonly used scores [minimum dietary diversity for children (MDD) and minimum dietary diversity for women] have been validated against nutrient adequacy in several contexts (4–8).

MDD is a core indicator in the WHO Global Nutrition Monitoring framework, and national estimates are tracked over time for all WHO member states (9). Many low- and middle-income countries (LMICs) have set national and subnational targets for MDD related to reducing the proportion of children with a low MDD or minimum acceptable diet (MAD), an indicator derived in part from MDD (10). Country profiles for the Global Nutrition Report, a global nutrition accountability report, include both MDD and MAD (11). Program evaluation guidance for USAID-funded programs also recommends MAD as an outcome indicator (12).

The global MDD indicator was first released by WHO-UNICEF in 2008 as part of a set of standard indicators to assess infant and young child feeding practices among children 0–23 months of age (2, 3). The original MDD indicator was tabulated using 7 food groups, including *1*) grains, roots, and tubers; *2*) legumes and nuts; *3*) dairy products; *4*) flesh foods; *5*) eggs; *6*) vitamin A–rich fruits and vegetables; and *7*) other fruits and vegetables (13). In 2017, MDD was revised to include breast milk as an eighth food group and is now defined as the proportion of infants and young children consuming least 5 of 8 food groups in 24 hours (9).

One of the challenges inherent in the use of simple dietary indicators to track population-level improvements in diet quality across time is that seasonal patterns often influence food availability, affordability, and accessibility in LMICs. Many countries experience seasonal lean seasons prior to the harvest, in which lower availability of food and money adversely affect access to more diverse and costly foods. Prices of nutrient-dense foods may also vary by season (14). Infants and young children may also experience seasonal patterns of illnesses, such as malaria, respiratory infections, and diarrhea, that adversely affect their appetite and the feeding practices used by parents (15).

Ideally, surveys used to generate national and subnational MDD estimates would be conducted at the same time each year to account for seasonality. However, numerous factors can prevent this from happening, such as political events and resource mobilization constraints. High-level monitoring of MDD across time in LMICs frequently relies on information collected through large-scale, multitopic household surveys. Necessary questions have been included in the Demographic and Health Survey's (DHS's) core questionnaire since round 6 (2009) and are included in the UNICEF Multiple Indicator Cluster Survey core questionnaire. Multiple countries in sub-Saharan Africa also implement annual national and subnational nutrition Standardized Monitoring and Assessment of Relief and Transitions (SMART) surveys that measure MDD. Large surveys are frequently conducted over several months and may span different seasons, with possibilities of variation in the timing of data collection by survey wave, resulting in estimated annual trends that are potentially influenced by seasonal patterns.

However, few analyses have examined the influence of seasonality on high-level estimates of dietary diversity indicators and none, to our knowledge, have explored the possible implications of the timing of data collection for global and national monitoring of progress on MDD over multi-year periods. In fact, a recent review of the seasonality of different causes of malnutrition in South Asia identified only 1 study examining the seasonality of child diets, and the study predated the development of the MDD indicator (16).

Using multi-year data sets collected across seasons in 3 countries with different child diets and seasonal patterns (Nepal, Peru, and Senegal) our study aims were to:

1. Examine the degree to which variability in the season of data collection may influence the national prevalence of MDD across survey years in 3 countries.
2. Understand the relative importance of seasonal compared with annual changes in this indicator.
3. Explore interactions between the seasonality of dietary diversity and rural/urban residence.

## Methods

### Description of data sources

Three different data sources were used for these analyses. Continuous DHS data sets were used for Senegal and Peru. For Nepal, data were used from a set of seasonal sentinel site surveys administered as part of the Policy and Science for Health, Agriculture, and Nutrition (PoSHAN) community studies. Across the 3 countries, we retained households with children 6–23 months of age having the available dietary data needed to construct the MDD indicator.

### Peru

The continuous DHS in Peru was the first DHS to collect data at multiple time points in a year and across consecutive years, and its methods are described in detail elsewhere (17). This analysis uses data from January 2005 to December 2016, available from the DHS Program and Peru National Institute of Statistics websites (18, 19). In brief, the survey used a 2-stage, household-stratified, cluster-sampling method and employed a cross-sectional design. The sample was stratified by urban/rural areas within 25 regions (Peru's 24 departments and 1 other province) and was conducted over a 10-mo period. The Peru continuous DHS was nationally representative starting in 2004 and also representative at the department level from 2008 onwards. Our analysis accounted for the 2-stage stratified-sampling procedure and changes in the sampling design (i.e., changes in survey strata) across years by adjusting for year-specific, stratified child weights. Sample sizes (number of children 6–23 months of age) are listed by month and year in **Supplementary Table 1**.

### Senegal

Data from the continuous DHS in Senegal from 2012–2017 were accessed from the DHS Program website (18). The DHS used a 2-stage, household-stratified, cluster-sampling method and also employed a cross-sectional design. The sample was nationally representative for all years and stratified by urban/rural areas within the 14 regions of Senegal. Detailed descriptions of the methods used are reported elsewhere (20, 21). However, as with the continuous DHS in Peru, the survey strata changed partway through the continuous survey in Senegal, in 2013. Our analysis again accounts for both the 2-stage stratified-sampling procedures and the changes in strata by adjusting for year-specific, stratified child weights. Sample sizes ranged from 772 in 2004 to 6404 in 2016, with a total of 12,183 children over the surveyed period. Sample sizes are presented by month and year in **Supplementary Table 2**. Data from 2012 and 2013 were combined into a single year (labeled 2013) in all analyses, as both represented half a year of continuous data from different seasons.

### Nepal

The Nepal data set included data on 1309 unique dietary assessments from 615 children aged 6–23 mo. The methods for collecting the seasonal data from PoSHAN Community Studies of Nepal are described in detail elsewhere (22–24). In brief, the larger PoSHAN study involved the collection of national data from 2013–2016, using a 2-stage, systematic, random sampling of 7 village development committees (VDCs) in each of Nepal's 3 agro-ecological zones: the mountains, hills, and *Tarai* (plains). Subsequently, 3 wards (the smallest administrative unit in Nepal) were randomly chosen per VDC (22). Within each of these wards, all consenting households with children <60 months of age and all consenting households with recently married couples without children were surveyed. Within this larger sampling frame of 21 VDCs and 63 wards, 1 VDC was selected in each zone as a sentinel site. These 3 sentinel sites were chosen based on their representativeness of the zone according to multiple indicators from the Central Bureau of Statistics (22).

Starting in 2013, households in the sentinel sites were prospectively followed across seasons for 2 continuous years, with data collected 3 times per year: in the monsoon lean season, during which rice and most other crops are grown (June/July/August); in the postharvest season (late August to September); and in the winter lean season (January/February). These 3 seasonal categories are consistent with those typically used in the literature for South Asia (16, 25). Additionally, data were collected during the 2016 monsoon season. Thus, the survey covers 7 time periods: 2 postmonsoon seasons (2013 and 2014), 2 winter seasons (2014 and 2015), and 3 monsoon seasons (2014, 2015, and 2016). Both cross-sectional and longitudinal analyses are possible, as households and children were tracked over time using longitudinal identifiers. New children could enter the seasonal sample surveys only during the monsoon seasons in 2014 and 2016. The result is that the average child was seen 2.1 times. Of the sample children, 45%, 21%, 13%, 21%, and 1% were viewed 1, 2, 3, 4, and 5 times, respectively. Similarly, given that we restricted the analysis to children 6–23 mo old, many children enrolled in 2013 had aged out by 2014, and all of them had aged out by 2016. Sample sizes for each period are presented in **Supplementary Table 3**.

### Outcome definition

MDD was calculated for children 6–23 months of age using standard classifications generated from a report produced by the WHO and UNICEF (26). Food groups included breast milk, grains/roots/tubers, legumes/nuts, dairy products, flesh foods, eggs, provitamin A–rich fruits and vegetables, and other fruits and vegetables. A child was defined as achieving MDD if they consumed at least 5 of the 8 food groups in the past 24 hours, resulting in a binary outcome variable. We also generated a continuous Food Group Score (FGS) variable as a count variable ranging from 0–8 and representing the total number of food groups the child consumed, including breast milk.

For Nepal, a 24-hour FFQ was administered to the primary child caretaker to capture child consumption of 31 commonly consumed food items. These were then consolidated into the food groups noted above, except for breast milk. A 7-day recall was administered to assess the number of times breast milk was consumed; children with any consumption were counted as “yes” and contributed to the dietary diversity score, those with no consumption over 7 days were counted as “no.” In Peru and Senegal, the continuous DHSs contained questions about the consumption of 25 and 17 different food groups, respectively, in the last 24 hours. These were designed to capture the majority of foods consumed by children in each country. The DHSs also indicated whether the child in question was currently breastfeeding. The information on food groups and breastfeeding was then consolidated into the same 8 food groups mentioned above to create the FGS and MDD.

### Definition of seasons

In Nepal, we compared the prevalence of MDD and the mean FGS across the 3 seasons defined by the original PoSHAN sampling design, described above. We hypothesized that both would be lower in the monsoon-lean and/or winter-lean seasons, as compared to the postharvest season.

For Senegal and Peru, we used 2 different approaches to defining seasons. The first was informed by our primary goal of understanding how the timing of data collection within a calendar year might influence estimates of the MDD prevalence and mean FGS. To that end, we defined the “lean season” as the 3 sequential months of lowest dietary diversity, recognizing that large national surveys often take multiple months to complete. We then compared the MDD prevalence and mean FGS across these 3 mo (lean season) compared with the rest of the year (nonlean season). In Peru, the lean season corresponded to May–July and in Senegal it corresponded to April–June. For Senegal, our definition of the lean season differed from the conventional definition that relates to food availability (June–September), because this period was not found to be the period of lowest dietary diversity.

A secondary seasonal contrast for these 2 countries emerged from a hypothesis that vegetables and fruits may be less available during the dry season, potentially leading to lower dietary diversity during that season. This hypothesis was tested by comparing the MDD and FGS in the rainy season compared with the dry season. We defined the rainy season as October–April in Peru and July–September in Senegal, based on maps of rainfall patterns by month, presented in **Supplementary Figures 1–3**, that were created in R (R Core Team)using the Era-Interim data from the European Center for Medium-Range Weather Forecasting, during the period of 1978–2018.

### Analysis

All estimates for Peru and Senegal were weighted averages or weighted regressions, according to the 2-stage sampling scheme. Adjusting for changes in sampling stratification across pooled cross-sectional data is necessary for a valid inference. Our adjustment follows DHS protocols for a pooled cross-sectional analysis, adjusting for changes in both survey strata and probability sampling units (clusters) over the years. The resulting survey weights reflect the probability of being sampled in any given year (this probability naturally falls with the population size), allowing averages or statistics to retain their nationally representative status. While dietary diversity variables are known to vary by wealth and child age, we did not include such variables in our models because the explicit goals of our analyses were to investigate whether the prevalence of MDD varies by season to a degree that it could drive spurious variation across survey rounds over time. The 95% CIs for all plots reflect SEs clustered at the DHS cluster level (Peru and Senegal) or ward level (Nepal), and regression SEs are clustered at these same levels.

First, the prevalences of MDD and mean FGSs with 95% CIs were calculated, plotted, and visually inspected by month, by year, and for each season for all 3 countries. Next, we examined seasonality in dietary diversity indicators using ordinary least squares (OLS) regression. In all regressions, dummy variables for years were included to parse the effect of season compared with more general temporal changes. Analyses for Peru and Senegal were weighted according to the 2-stage sampling scheme, with errors clustered at the DHS cluster level. Simple OLS analyses for Nepal had errors clustered at the ward level. Additional OLS regressions for Nepal included child fixed effects (random intercepts) with errors clustered at the child level. This fixed-effect specification further isolates the role of seasonal variation by controlling for any unobserved, time-invariant child characteristics that influence dietary diversity; effectively, children are being used as their own controls when observed both during and outside of the season of interest. Student *t*-tests were used to test for seasonal differences in outcomes in all countries, and an F-test was used to test the joint influence of the 3 agricultural seasons in Nepal. In a last specification, we also examined an interaction between seasons and urban/rural residence, to explore whether seasonal patterns could differ across these strata, which represent very different food environments. For all regressions, tests of statistical significance are denoted at the 1%, 5%, and 10% levels. Data were analyzed in SAS 9.4 (SAS Institute Inc.), STATA 14.2 (Stata Statistical Software: Release 14; StataCorp LP), and R Studio 1.1383 running R 3.4.3 (R Core Team).

### Consent and institutional review board approvals

Ethical approval for the PoSHAN study in Nepal was granted by the institutional review board (IRB) at the Johns Hopkins Bloomberg School of Public Health (JHBSPH) and from the Nepal Health Research Council. Ethical oversight of the DHSs for Peru and Senegal is managed by the IRB at ICF International, and use of the data sets was deemed not to be human subjects research by the IRB at JHBSPH, as these are deidentified data sets.

## Results

The sample included 35,272 children aged 6–23 mo from Peru; 12,183 from Senegal; and 615 children, representing 1309 observations over time, from Nepal. The temporal distributions of children with MDD/FGS measures are presented in Supplementary Tables 1–3. These tables show the presence of certain data gaps for each of the data sets that were due to the design and implementation of multiple data collection rounds: for example, in Peru, the period from 2004 to 2008 had fewer observations for the months of October to December, while 2007 to 2016 had fewer observations from January and February. Similar gaps exist in the data for both Senegal and Nepal.

Selected characteristics of the 3 country samples are presented in **Table 1**. About one-third of the Peru sample was collected from rural areas, compared with about two-thirds of the Senegal sample. The Nepal sample was made up mainly of rural areas, although 1 ward was semiurban. Peru had the highest prevalence of MDD in the full sample, at about 75%, compared with about 10% in Senegal and 39% in Nepal.

**TABLE 1:** Characteristics of the data sets and populations

	Peru	Senegal	Nepal^1^
Number of children 6–23 months of age	35,272	12,183	1309
Rural, %	32.0	64.6	100
Food group score,^1^ mean (SD)	5.4 (1.87)	2.9 (1.54)	4.09 (0.29)
Minimum dietary diversity, % ≥ 5	75.4	15.7	39.1
HAZ, mean (SD)	–1.04 (1.014)	–0.89 (1.19)	–1.75 (0.08)
Stunting, HAZ < –2 z-scores, %	16.4	15.9	41.1
WHZ, mean (SD)	–0.44 (1.179)	–1.12 (1.12)	–0.96 (0.13)
Wasting, WHZ < -2 z-scores, %	8.0	21.7	15.4

All statistics are across sample children, not households. For Peru and Senegal, estimates are weighted. HAZ, height-for-age z-score; WHZ, weight-for-height z score.

1 In Nepal, the number of children represents the total number of measurements conducted among the 615 unique children.

**FIGURE 1:**
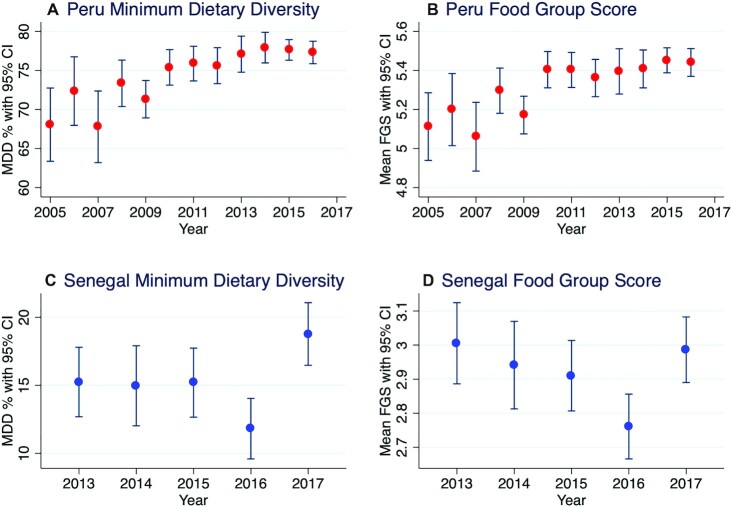
Prevalence of MDD and mean FGS by survey year in Peru and Senegal. FGS, Food Group Score; MDD, minimum dietary diversity for children.

**Figure 1** presents annual trends in estimates of the MDD prevalences and mean FGSs for Peru and Senegal at the national level. In Peru, the annual prevalence of MDD rose steadily between 2005 and 2013, where it then plateaued; the highest estimate of MDD prevalence was observed in 2014 at 77.9% (95% CI, 76.0%–79.9%). The mean FGS for Peru exhibited a similar rise through 2013, peaking in 2015 at 5.45 food units (95% CI, 5.39–5.52 food units). For Senegal, the annual prevalence of MDD stayed constant during 2013–2015 at ∼15%, fell in 2016 to just under 12%, then rose in 2017 to 18.8% (95% CI, 16.8%–20.7%). Interestingly, annual estimates of the mean FGS showed a fairly steady decline between 2013 and 2016 in Senegal, despite the fact that MDD stayed constant for the first 3 y. Then, like MDD, the mean FGS rose in 2017 to 3.0 food units (95% CI, 2.91–3.07 food units), almost exactly where it had started in 2013. Therefore, while a clear overall improvement was observed in dietary diversity in Peru, only minor year-to-year variations in dietary diversity were observed in Senegal, with very little (MDD) or no (FGS) overall improvement.

**FIGURE 2:**
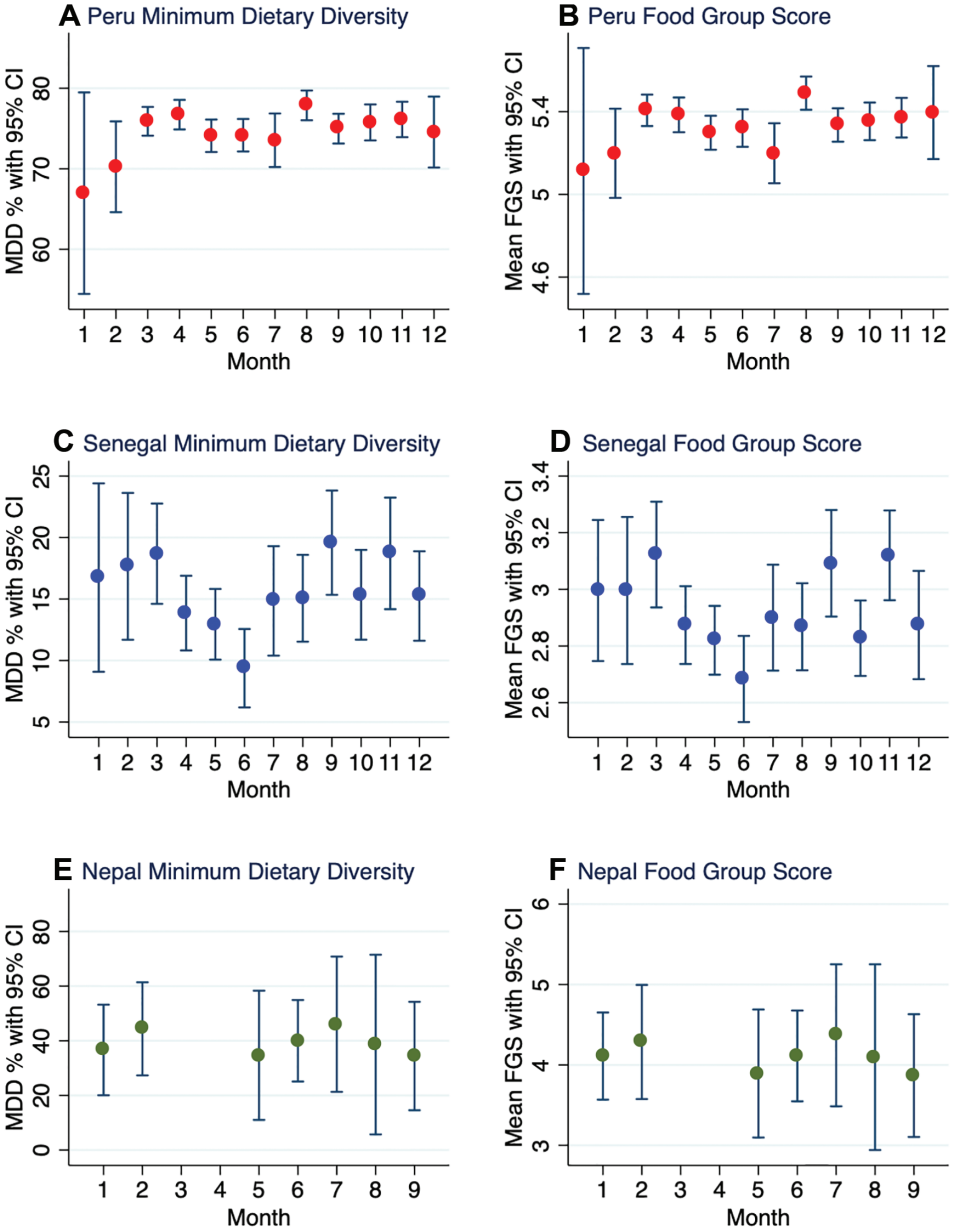
Prevalence of MDD and mean FGS by month for Peru, Senegal, and Nepal, with data pooled across survey years. FGS, Food Group Score; MDD, minimum dietary diversity for children.

**Figure 2** presents the prevalences of MDD and mean FGSs disaggregated by month, pooled across all years/data collection points for all 3 countries. The prevalences of MDD exhibited monthly variations in all countries. For Peru, a slight seasonal dip was observed from May to July, where the MDD prevalence hovered around 74%, followed by a rise in August. While a dip in the MDD prevalence was also observed in January and February, very few observations exist in those months, and so the CIs around those estimates are too wide to draw inferences about the true seasonal mean. Seasonality was much more pronounced in Senegal, where MDD prevalence estimates hovered between 15%–20% in all months except April–June, where they dropped below 15%. The MDD prevalence was lowest in June, at only 9.34% (95% CI, 6.2%–12.6%). In Nepal, the estimated MDD prevalence was bound within an approximate 12% point range across months, ranging from 34.7% (95% CI, 11.0%–58.3%) in May to 46.1% (95% CI, 21.3%–70.8%) in July. However, some months did not have data collection activity, and so it is possible that this range of 12% could be larger if the data collection period had spanned all months of the year. CIs were also widest in Nepal, due to the relatively smaller sample size of this survey.

**FIGURE 3:**
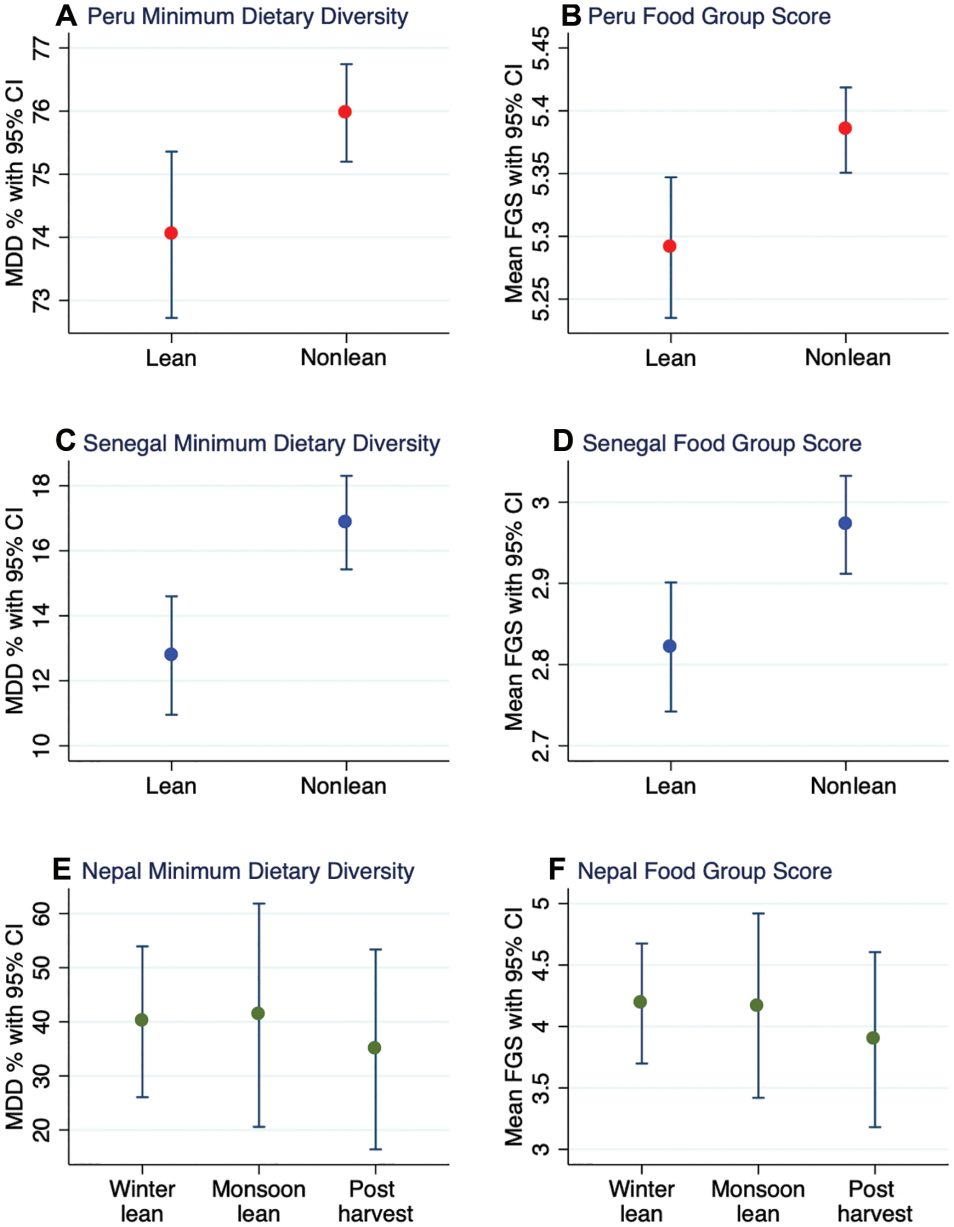
Prevalence of MDD and mean FGS, disaggregated by lean seasons in Peru, Senegal, and Nepal. FGS, Food Group Score; MDD, minimum dietary diversity for children.

In Peru and Senegal, the national prevalences of MDD were 1.9% and 4.1% lower, respectively, in the lean season than the rest of the year. In both countries, the mean FGS was about 0.1 units lower in the lean season than during the rest of the year (**Figure 3** ). In Nepal, the postharvest season had a lower seasonal estimate than the other 2 seasonal data points, although all estimates were bound by wide and overlapping 95% CIs. For both Peru and Senegal, MDD prevalences and mean FGSs were similar for the rainy and nonrainy seasons (**Supplementary Figure 4**).

**FIGURE 4:**
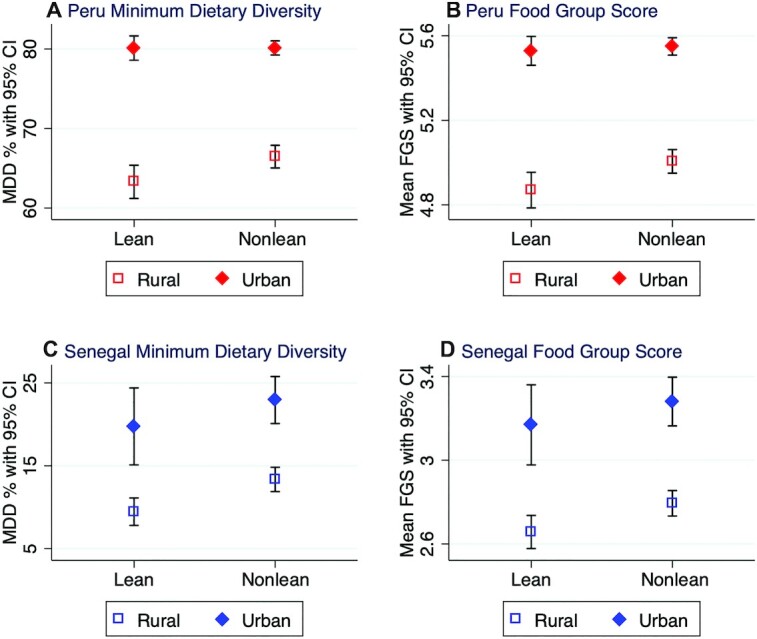
Prevalence of MDD and mean FGS, disaggregated by lean season and rural/urban locality, in Peru and Senegal. FGS, Food Group Score; MDD, minimum dietary diversity for children.

Using a regression analysis to separate seasonal variation from year-by-year variation, we continued to find evidence of small but consistent seasonal patterns, suggesting that dietary diversity was lower in the lean season. Analyses of national data sets from Peru and Senegal suggested the child MDD prevalences were 1.8% and 3.5% lower, respectively, during the lean season (**Table 2**, columns 1 and 3). A simple OLS analysis suggests that in Nepal, the child MDD was similarly 1.3% lower during the lean season (Table 2, column 5), and a child fixed-effects analysis showed similar results (**Supplementary Table 4**). However, CIs were much wider in Nepal due to the smaller sample size (11% and 4% of the Peru and Senegal sample sizes, respectively), and thus coefficients were not statistically significant. For FGSs, in all 3 countries, children consumed about a tenth of a food group less during the lean season, although the findings were statistically significant (*P *< 0.01) only in Peru and in Senegal (Table 2). No significant differences were observed in Peru or Senegal for the rainy compared with the dry season (**Supplementary Table 5**).

**TABLE 2:** Minimum dietary diversity and Food Group Scores by season

	Peru^1^^,^^2^		Senegal^1^^,^^2^		Nepal^1^^,^^3^	
	MDD	FGS	MDD	FGS	MDD	FGS
	*n* = 35,272	*n* = 35,272	*n* = 12,183	*n* = 12,183	*n* = 1309	*n* = 1309
Lean season	–0.0179^4^ (–0.033 to –0.002)	–0.087^4^ (–0.154 to –0.021)	–0.0347^5^ (–0.058 to –0.011)	–0.140^5^ (–0.245 to –0.035)	–0.0133 (–0.107 to 0.081)	–0.117 (–0.528 to 0.293)
Post-harvest	—	—	—	—	–0.0139 (–0.130 to 0.102)	–0.123 (–0.467 to 0.222)
R^2^	0.004	0.004	0.006	0.005	0.010	0.019
Joint F-test *P* value					0.938	0.692

DHS, Demographic Health Survey; FGS, Food Group Score; MDD, minimum dietary diversity for children; OLS, ordinary least squares.

1 All models were fit using OLS; year fixed effects were included in all models.

2 SEs are clustered by DHS cluster. The table presents 95% CIs.

3 Nepal data were fit with simple OLS with SEs clustered by cluster. The lean season represents the monsoon lean season. The monsoon lean and postharvest seasons are tested against the omitted winter lean season, and the F-test *P* value at the bottom of the table tests the joint significance of both seasons.

4 *P *< 0.05.

5 *P *< 0.01.

In regression models testing for the interaction of urban/rural residence and the lean season, significant seasonal patterns in both the MDD and FGS were apparent in the rural areas of Senegal and Peru, but not in urban areas (**Table 3**). In both countries, the prevalence of MDD fell by 3% during the lean season in rural areas (**Figure 4**). Both the prevalence of MDD and the mean FGS were also higher in urban households, who consumed half a food group more than rural households on average. Both the prevalence of MDD and the mean FGS were marginally higher during the rainy season in rural areas (**Supplementary Table 6)**, even though little difference was observed in the rainy season at the national level or in urban areas. We also explored the possibility that differences in the timing of visits by survey teams to different regions in Peru and Senegal might be responsible for observed seasonality by adding regional fixed effects to these rural/urban regressions (**Supplementary Table 7**). Results did not meaningfully differ from those in Table 3, suggesting that regional differences in survey implementation timing are not responsible for seasonality.

**TABLE 3:** Minimum dietary diversity and Food Group Scores by season and rural/urban locality in Peru and Senegal

	Peru^1^^,^^2^		Senegal^1^^,^^2^	
	MDD	FGS	MDD	FGS
	*n* = 35,272	*n* = 35,272	*n* = 12,183	*n* = 12,183
Lean season,^3^ rural	–0.0296^4^ (–0.055 to –0.004)	–0.126^4^ (–0.228 to –0.024)	–0.0309^5^ (–0.054 to –0.008)	–0.127^4^ (–0.230 to –0.024)
Lean season,^3^ urban	–0.000210 (–0.018 to 0.018)	–0.0193 (–0.099 to 0.061)	–0.0298 (–0.082 to 0.022)	–0.103 (–0.324 to 0.118)
Urban	0.134^5^ (0.117–0.151)	0.535^5^ (0.465–0.604)	0.0971^5^ (0.066–0.128)	0.486^5^ (0.358–0.614)
R^2^	0.028	0.023	0.022	0.028

DHS, Demographic Health Survey; FGS, Food Group Score; MDD, minimum dietary diversity for children; OLS, ordinary least squares.

1 All models fit using OLS; year fixed effects were included in all models.

2 Standard errors are clustered by DHS cluster. Table presents 95% CIs.

3 *P *< 0.1.

4 *P *< 0.05.

5 *P *< 0.01

**FIGURE 5:**
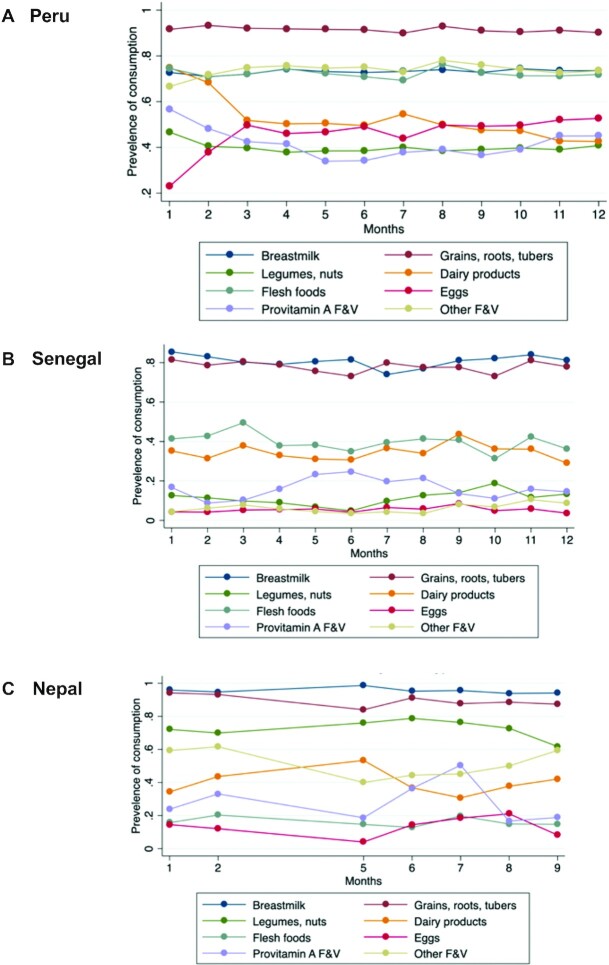
Prevalence of consumption of specific MDD components by month for Peru, Senegal, and Nepal. F&V, fruits and vegetables; MDD, minimum dietary diversity for children.

The proportion of children consuming individual food groups was also explored in each country (**Figure 5**). Results from Peru suggested that the consumption of most food groups was stable around the year. In Senegal, breast-milk consumption was negatively correlated with staple consumption. Additionally, we see that while consumption of both flesh foods and provitamin A–rich foods dropped during August–October, consumption of legumes and nuts rose during this same period, suggesting a buffering effect. These trends were even stronger in rural areas. In Nepal, the rise in FGS in July appeared driven by a rise in provitamin A–rich fruits and vegetables and in eggs, though this rise was accompanied by a decrease in dairy consumption in July. In Senegal, a similar inverse relationship to that in Peru was observed between breast-milk consumption and staple consumption.

## Discussion

This study helps to fill an important gap in the measurement agenda related to the use of the MDD indicator for high-level monitoring of the quality of diets of infants and young children in LMICs. Much of the validation work related to the child MDD indicator to date has focused on its ability to serve as a proxy for nutrient adequacy (27, 28). Subsequently, refinement of the indicator from a 7-item to an 8-item score including breast milk took place to improve its utility as a more comprehensive indicator of children's diets during the 6- to 23-mo period (10, 11). However, even though patterns in food insecurity and food availability are known to be seasonal in many rural contexts, we believe that this is the first study to examine the seasonality of the MDD indicator from the perspective of understanding its validity for monitoring high-level changes in diet quality over time (i.e., the type of monitoring done by UN agencies, governments, and global accountability initiatives).

Despite the vastly different prevalence of MDD in the 3 contexts, remarkable similarities were observed in the magnitude of lean season effects on MDD. Although the seasonal patterns were strongest in Peru, the range of lean season coefficients for MDD across the 3 settings was fairly consistent, ranging from 2% to 4%. Even greater consistency was observed across all 3 countries in the magnitude of seasonal effects on the FGS indicator. In both Peru and Senegal, no significant seasonality in either MDD or FGS was present for children in urban areas.

Growing attention is being paid to country-level progress related to dietary shifts over time, and the MDD represents a key indicator for many countries and agencies, as it is easily collected and can be included in major, large-scale surveys such as the DHS and Multiple Indicator Cluster Surveys. These surveys are often spaced at 5-y intervals or longer, with the change in prevalence of sequential surveys used to infer trends. Some countries, including multiple countries in the West Africa region, conduct annual nutrition surveys between DHS and MICS years and infer MDD trends over a shorter interval. Yet the timing of such surveys within a calendar year is not necessarily calibrated to occur within the same months or season, as many factors may influence the timing of a survey going to the field.

Our research aimed to examine whether seasonal differences in the timing of surveys might exert a spurious influence on inferences about national-level changes in MDD over longer periods of time, particularly if 2 surveys were not conducted in the same season. Often the period between national surveys is 5 y. Extrapolating the observed annual rates of change for MDD in Peru and Senegal per 5 y (by subtracting the last annual estimate from the first, dividing by the observation period, and multiplying by 5), we observed estimated MDD increases by an average of 4.2 percentage points for Peru and 4.4 percentage points for Senegal per 5 y. These multi-year changes were not markedly larger than the average seasonal changes in MDD observed in these countries, which ranged from 2–4 percentage points, as noted above. This suggests that the seasonal variability in MDD prevalences could indeed be large enough to introduce bias into long-term MDD trends and to lead to the wrong conclusions. The same was true for the FGS; the average 5-y rate of change in FGSs was 1.15 food items in Peru (an improvement) and 0.02 food items in Senegal (a slight decline). Yet FGSs varied across the lean compared with nonlean seasons by 0.09 items in Peru and 0.14 items in Senegal, again suggesting that seasonality may introduce spurious influences on multi-year estimates of trends in dietary diversity, particularly in countries like Senegal that are experiencing slow change.

However, governments and large-scale projects often assume that much greater improvements in dietary diversity will be made over time than those we observed in the present study. For example, the nutrition-sensitive agriculture strategy in Ethiopia set an ambitious target for change in the MDD indicator from 9.8% to 40% from 2016 to 2020, with planned annual surveys to detect a change of approximately 6% per year (29). The seasonal fluctuations in data collection across years observed in our study would exert only a minor influence on long-term prevalence estimates in countries where such rapid rates of change are occurring, but might still be important to consider if annual measurements were being compared. For many countries, particularly those with a higher baseline MDD prevalence, the expected change between years would be much less, and even small seasonal fluctuations of the magnitude we observed could influence inferences about progress over time.

Our study also revealed some insights into the comparative responsiveness of MDD and FGS to seasonality. Dichotomizing an indicator (such as FGS into MDD) results in the loss of information, which is particularly important when considering changes in indicators over time (e.g., across rounds of national surveys) or space (e.g., across regions within a country). The MDD measure will only reflect a change in dietary diversity when the threshold of FGS ≥5 is crossed. So, if dietary diversity is improving amongst segments of the population with much lower or higher mean FGSs, that change may not be captured by the MDD measure. This helps to explain why the drop in the mean FGS experienced in Senegal between 2013 and 2015 was not mirrored by an accompanying drop in the MDD prevalence. Because the average FGS is low in Senegal (2.9 over the entire survey period), changes in FGS are not necessarily reflected in changes in MDD, because they happen below the FGS threshold of 5.

This study responds to calls for further validation of the seasonal dimensions of dietary diversity scores, and is the first to our knowledge to explore the seasonality of the MDD indicator among children 6–23 months of age. Surprisingly few studies have examined the seasonality of diets of infants and young children in global settings. A recent comprehensive review of seasonal determinants of undernutrition in South Asia identified just 1 study, from 1985, exploring seasonality of dietary intakes among infants in Bangladesh, which predated development of the MDD indicator (15, 16). A recent study conducted among 6- to 59-mo-old children and their mothers from Timor-Leste found evidence of lower dietary diversity during the dry and wet seasons compared with the dry and transition seasons, although a lower threshold of only 2 food groups was used to define low dietary diversity compared with the MDD indicator (30).

However, a number of studies have been conducted among older children using different indicators, including more detailed, quantified measures of intakes. A study among older preschool-aged children (36–59 months of age) in Burkina Faso using 24-h recall methods found slightly greater dietary diversity in the postharvest season (3.88/7 food groups) compared with the lean season (3.64 food groups), despite finding fairly notable differences in nutrient adequacy of 10 nutrients (0.52 compared with 0.43) (31). Given the study population consisted of older children, breast milk was not included, and it is possible that the inclusion of breast milk could attenuate seasonal effects in an 8-item scale, particularly in rural settings with high breastfeeding rates. In a population of school-aged children 4–8 years of age in rural Zambia, researchers found that seasonality was predictive of a 7-item score and also influenced the ability of the dietary diversity score to capture nutrient adequacy (32). In rural Tanzania, researchers found no difference in 1-d or 7-d mean dietary diversity scores among a population of 2- to 5-y-old children, but found a slight difference in 1-d food variety scores, driven by the use of wild/forest foods during the wet, food-insecure season (33).

Lack of seasonality of the FGS and certain components (i.e., specific food groups) could be explained in part by a buffering effect, as different items in a food group become available across different seasons. For example, provitamin A–rich orange fruits, such as mangoes, often have widespread availability during a very limited season in many countries (30, 34). In some cultures, fruits are also perceived to be more of a child's food, which could strengthen seasonal patterns of consumption in this age group (33). During seasons of food shortages, households may also buffer the effects of seasonality by preferentially allocating food to children, as observed in a study from Benin (35). It is also possible that items within a food group may change over time, minimizing variability around the year; for example, green leafy vegetables are grouped into the same category as orange-fleshed fruits, and so lack of availability of orange-fleshed fruits in some seasons could be buffered by greater availability of green leafy vegetables. Wild greens are often locally available across the year in the food system, and poorer households may rely on them more (35). Such effects could help to explain why for most food groups we saw little fluctuation by month in the contexts that we examined. Because flesh foods are seldom consumed in many contexts, it would be expected that variation is largely random. However, flesh-food consumption could be higher in festival seasons, which vary by country in terms of the consistency of their timing. Breast milk may be a stable source of food across seasons, although there is some evidence of slight seasonality to consumption patterns (15). There was some evidence that inversely correlated seasonal variations across different food groups may act to cancel each other out, in Senegal in particular, and moreover that breast milk may act as a buffer for staples in particular in both Nepal and Senegal.

Other plausible explanations exist for the lack of strong seasonal patterns observed for MDD and FGS measures in our study. Nepal and Peru have some of the greatest variations in altitude and agro-ecological zones in the world. If different parts of the country are following different seasonal patterns, there could be a smoothing of seasonal effects at the national level. Our observation that seasonal patterns were much stronger in rural than urban areas lends support to this argument. Another plausible explanation for lack of strong seasonality is that the indicator simply measures consumption of each item within the past day, not the amount consumed. If foods are consumed in lesser amounts during the lean season but still consumed, as observed for meat and fish in Benin (35), dietary diversity indicators may not be responsive to such changes. If detecting seasonal changes over time is a measurement priority, the use of measurement approaches that incorporate quantities of foods consumed could be important.

Our study also revealed that the diets of children living in urban areas of both Peru and Senegal are more diverse than the diets of children in rural areas of both countries, a finding that is consistent with the literature (36). However, because urban populations tend to purchase rather than grow their foods, access to healthier (and more seasonal) foods, such as fruits and vegetables, tends to be strongly tied to socio-economic status. While we did not detect the presence of seasonal effects on the diets of children in urban Peru or Senegal, further work might explore whether such effects might exist within specific socioeconomic subgroups of urban children.

An important limitation to our study was that the Nepal sample was much smaller than the Peru and Senegal samples. It was also drawn from a smaller geographical area (9 wards total from 3 zones, compared with national samples), limiting the external generalizability of findings. The small sample size resulted in wide CIs around estimates and likely underpowered tests of statistical significance for seasonal effects. However, multiple observations taken from the same children, households, and areas over time in Nepal and the near-complete enumeration of these areas allowed us to better control for external sources of variation and to minimize sampling errors.

In conclusion, we observed the presence of seasonal patterns in MDD in Peru and Senegal, but our sample size for Nepal likely limited the ability to detect seasonality. The magnitude of seasonal effects appeared smaller than we initially expected, but was large in relation to longer-term trends. If large-scale surveys were conducted in different seasons, the seasonal effects could be large enough to lead to incorrect inferences about change over time. We recommend that whenever possible, the DHS, MICS, and locally planned national nutrition surveys be conducted in the same season over time. When they are not, the timing of surveys relative to the season should be considered when interpreting trends. While our work sheds some light on the seasonal patterns of MDD in 3 very different contexts, additional work is needed in other settings to explore how seasonality influences this and other dietary diversity indicators (2). Further investigation is also needed to understand the potential role of region–season interactions in attenuating seasonal shifts in national estimates of MDD measures in Peru and Senegal.

## Supplementary Material

nzab095_Supplemental_File

## Notes

Support for this research was provided by the Bill & Melinda Gates Foundation, Seattle, WA, and the Feed the Future Innovation Lab for Nutrition, which is funded by the United States Agency for International Development under grant number AID-OAA-L-1-00006.

Author disclosures: The authors report no conflicts of interest.

The funders did not play a role in the design, implementation, analysis, or interpretation of the data.

Supplemental Figures 1–4 and Supplemental Tables 1–7 are available from the “Supplementary data” link in the online posting of the article and from the same link in the online table of contents at https://academic.oup.com/cdn/.

Abbreviations used: DHS, Demographic Health Survey; FGS, Food Group Score; IRB, institutional review board; JHBSPH, Johns Hopkins Bloomberg School of Public Health; LMIC, low- and middle-income country; MAD, minimum acceptable diet; MDD, minimum dietary diversity for children; OLS, ordinary least squares; PoSHAN, Policy and Science for Health, Agriculture, and Nutrition; VDC, village development committee.
