# Supplementary material for: Season of Data Collection of Child Dietary Diversity Indicators May Affect Conclusions About Longer-Term Trends in Peru, Senegal, and Nepal
Source: Curr Dev Nutr. 2021 Jul 12;5(8):nzab095. doi: 10.1093/cdn/nzab095 (PMC8397594; doi:10.1093/cdn/nzab095)
Supplement: nzab095_Supplemental_File [file nzab095_supplemental_file.docx]

**Online Supplementary Material**

**Supplementary Table 1. Sample size by month and year for the Peru DHS sample (6-23 month old infants and young children)**

| Year | Month of interview | | | | | | | | | | | | |
| --- | --- | --- | --- | --- | --- | --- | --- | --- | --- | --- | --- | --- | --- |
|  | 1 | 2 | 3 | 4 | 5 | 6 | 7 | 8 | 9 | 10 | 11 | 12 | Total |
| 2005 | 35 | 103 | 96 | 130 | 78 | 90 | 80 | 113 | 116 | 1 | 0 | 0 | 842 |
| 2006 | 105 | 104 | 79 | 79 | 114 | 86 | 118 | 135 | 76 | 0 | 0 | 0 | 896 |
| 2007 | 2 | 106 | 115 | 33 | 114 | 109 | 95 | 92 | 117 | 3 | 0 | 0 | 786 |
| 2008 | 0 | 74 | 346 | 417 | 372 | 217 | 106 | 158 | 152 | 90 | 0 | 0 | 1932 |
| 2009 | 0 | 0 | 340 | 428 | 437 | 335 | 15 | 419 | 450 | 478 | 276 | 1 | 3179 |
| 2010 | 0 | 0 | 415 | 335 | 363 | 259 | 7 | 284 | 362 | 362 | 386 | 48 | 2821 |
| 2011 | 0 | 0 | 323 | 329 | 390 | 320 | 24 | 296 | 306 | 360 | 284 | 36 | 2668 |
| 2012 | 0 | 0 | 334 | 388 | 316 | 320 | 95 | 289 | 297 | 321 | 291 | 201 | 2852 |
| 2013 | 0 | 0 | 293 | 307 | 286 | 272 | 115 | 241 | 359 | 310 | 306 | 199 | 2688 |
| 2014 | 0 | 0 | 290 | 309 | 388 | 310 | 202 | 511 | 346 | 315 | 167 | 51 | 2889 |
| 2015 | 0 | 0 | 714 | 829 | 859 | 817 | 362 | 818 | 894 | 866 | 832 | 324 | 7315 |
| 2016 | 0 | 0 | 654 | 670 | 736 | 689 | 354 | 670 | 774 | 823 | 764 | 270 | 6404 |
| Total | 142 | 387 | 3999 | 4254 | 4453 | 3824 | 1573 | 4026 | 4249 | 3929 | 3306 | 1130 | 35272 |

**Supplementary Table 2. Sample size by month and year for the Senegal DHS sample (6-23 month old infants and young children)**

| Year | Month | | | | | | | | | | | | |
| --- | --- | --- | --- | --- | --- | --- | --- | --- | --- | --- | --- | --- | --- |
|  | 1 | 2 | 3 | 4 | 5 | 6 | 7 | 8 | 9 | 10 | 11 | 12 | Total |
| 2012 | 0 | 0 | 0 | 0 | 0 | 0 | 0 | 0 | 53 | 196 | 245 | 277 | 771 |
| 2013 | 257 | 0 | 271 | 302 | 342 | 163 | 0 | 0 | 0 | 0 | 0 | 0 | 1335 |
| 2014 | 42 | 257 | 340 | 327 | 52 | 254 | 231 | 190 | 231 | 144 | 0 | 0 | 2068 |
| 2015 | 0 | 221 | 287 | 307 | 312 | 1 | 133 | 371 | 248 | 253 | 14 | 0 | 2147 |
| 2016 | 0 | 0 | 199 | 352 | 364 | 122 | 96 | 342 | 207 | 308 | 115 | 0 | 2105 |
| 2017 | 0 | 0 | 1 | 206 | 478 | 10 | 552 | 497 | 417 | 619 | 544 | 433 | 3757 |
| Total | 299 | 478 | 1098 | 1494 | 1548 | 550 | 1012 | 1400 | 1156 | 1520 | 918 | 710 | 12183 |

**Supplementary Table 3. Sample size^1^ by round for the Nepal seasonal sample (6-23 month old infants and young children)**

|  | Sample size^1^ |
| --- | --- |
| End monsoon harvest, 2013 | 168 |
| Winter lean, 2014 | 139 |
| Monsoon, 2014 | 203 |
| End monsoon harvest, 2014 | 193 |
| Winter lean, 2015 | 166 |
| Monsoon, 2015 | 230 |
| Monsoon, 2016 | 210 |

**^1^**# of unique measures

**Supplementary Table 4. Minimum dietary diversity and food group score by season in Nepal, with child fixed effects**

|  | Child Fixed Effect OLS^1^ | |
| --- | --- | --- |
|  |  |  |
|  | MDD-C | FGS |
|  | N=1312 | N=1312 |
| Monsoon lean season | -0.0156 (0.0313) | -0.0896 (0.0908) |
| Post-harvest season | 0.0084 (0.0438) | 0.0484 (0.127) |
|  |  |  |
| Observations | 1,312 | 1,312 |
| R^2^ |  |  |
| Within-child R^2^ | 0.0581 | 0.134 |
| Joint F-test p-value | 0.772 | 0.345 |

^1^Models fit using ordinary least squares (OLS) with year fixed effects, with standard errors clustered by child in parentheses. Monsoon lean and postharvest season are tested against omitted winter

lean season (Pearson’s T); no differences are significant. An F-tests tests the joint significance

of the winter lean and postharvest season; no differences are significant.

*p<0.1, **p<0.05, ***p<0.01

**Supplementary Table 5. Minimum dietary diversity and food group score by rainy season in Peru and Senegal**

|  | Peru^1,2^ | |  | Senegal^1,2^ | |
| --- | --- | --- | --- | --- | --- |
|  | MDD | FGS |  | MDD | FGS |
|  | N=35,996 | N=35,996 |  | n=12,183 | N=12,183 |
| Rainy season | 0.0118 (0.00754) | 0.0481 (0.0282) |  | 0.0184 (0.0119) | 0.0878 (0.0562) |
| R^2^ | 0.008 | 0.005 |  | 0.006 | 0.003 |

^1^All models fit using ordinary least squares (OLS) with year fixed effects included in all models.

^2^Standard errors are clustered by DHS cluster.

*p<0.1, **p<0.05, ***p<0.01

**Supplementary Table 6. Minimum dietary diversity and food group score by rainy season and urban vs. rural location for Peru and Senegal**

|  | Peru^1,2^ | |  | Senegal^1,2^ | |
| --- | --- | --- | --- | --- | --- |
|  | MDD | FGS |  | MDD | FGS |
|  | N=35996 | N=35996 |  | n=12183 | N=12183 |
| Rainy season*rural | 0.0225* (0.0117) | 0.0987** (0.0430) |  | 0.0244** (0.0106) | 0.0815 (0.0562) |
| Rainy season*urban | 0.00283 (0.00919) | 0.00972 (0.0347) |  | 0.000945 (0.0235) | 0.0562 (0.101) |
| Urban | 0.151*** (0.0103) | 0.558*** (0.0380) |  | 0.0793*** (0.0123) | 0.397*** (0.0570) |
| R^2^ | 0.027 | 0.023 |  | 0.019 | 0.02 |

^1^All models fit using ordinary least squares (OLS); year and region fixed effects included in all models.

^2^Standard errors are clustered by DHS cluster. Table presents standard errors.

*p<0.1, **p<0.05, ***p<0.01

**Supplementary Table 7. Minimum dietary diversity and food group score by season and urban vs. rural location in Peru and Senegal, with region fixed effects**

|  | Peru^1,2^ | |  | Senegal^1,2^ | |
| --- | --- | --- | --- | --- | --- |
|  | MDD | FGS |  | MDD | FGS |
|  | N=35996 | N=35996 |  | n=12183 | N=12183 |
| Lean season*rural | -0.0322***  [-0.056,-0.009 ] | -0.108**  [-0.194,-0.022] |  | -0.0235***  [-0.039,-0.008] | -0.0980**  [-0.186,-0.010] |
| Lean season*urban | -0.0147  [-0.035, 0.007] | -0.0595  [-0.136, 0.017] |  | -0.00450  [-0.041, 0.050] | -0.0382  [-0.238, 0.162] |
| Urban | 0.132***  [0.114, 0.150] | 0.472***  [0.405, 0.540] |  | 0.0302***  [0.007, 0.053] | 0.160***  [0.049, 0.272] |
| R^2^ | 0.042 | 0.039 |  | 0.030 | 0.038 |

^1^All models fit using ordinary least squares (OLS); year and region fixed effects included in all models.

^2^Standard errors are clustered by DHS cluster. Table presents 95% confidence intervals.

*p<0.1, **p<0.05, ***p<0.01

**
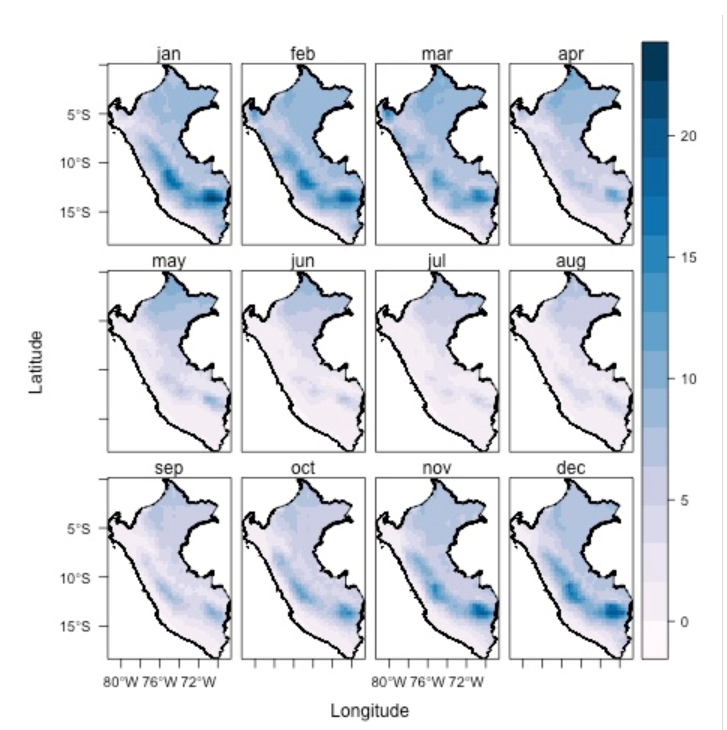
**

**Supplementary Figure 1. Rainfall patterns by month in Peru**

**
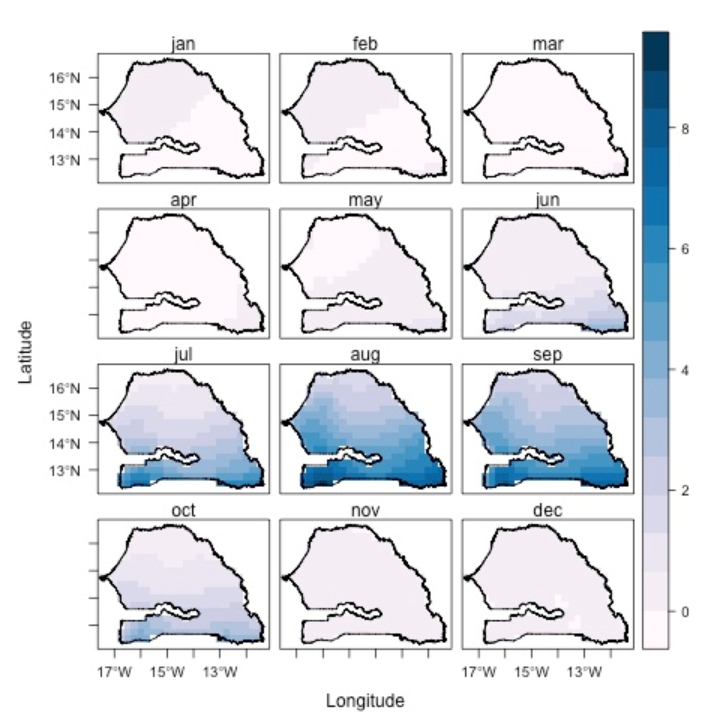
**

**Supplementary Figure 2. Rainfall patterns by month in Senegal**

**
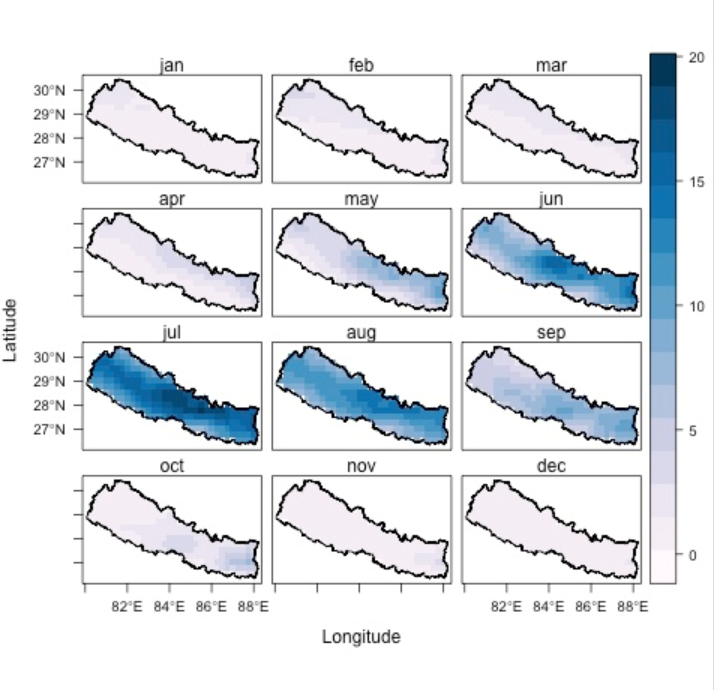
**

**Supplementary Figure 3. Rainfall patterns by month in Nepal**

**
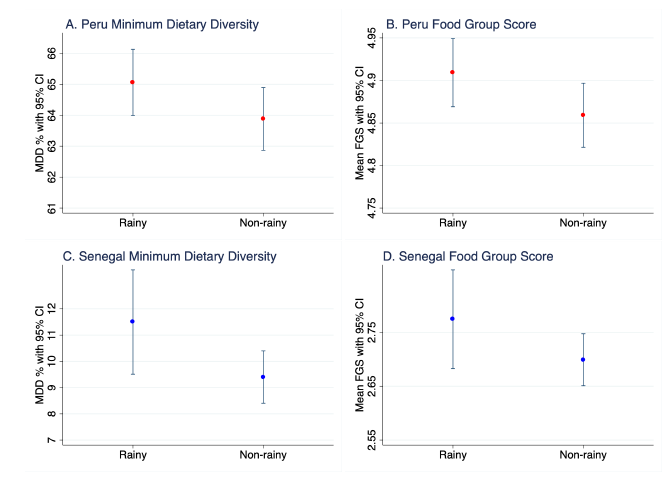
**

**Supplementary Figure 4. Prevalence of MDD and FGS by rainy season, pooled across years**
